# Characterization of a cotton-wool like composite bone graft material

**DOI:** 10.1007/s10856-022-06682-3

**Published:** 2022-07-18

**Authors:** Nadja Rohr, Claudia Brunner, Benjamin Bellon, Jens Fischer, Michael de Wild

**Affiliations:** 1 Biomaterials and Technology, Clinic for Reconstructive Dentistry, University Center for Dental Medicine Basel, Basel, Switzerland; 2 Private Practice, Oberentfelden, Switzerland; 3 Department of Preclinical and Translational Research, Institut Straumann AG, Basel, Switzerland; 4 School of Life Sciences, Institute for Medical Engineering and Medical Informatics IM², University of Applied Sciences Northwestern Switzerland, Muttenz, Switzerland

**Keywords:** Bonewool^®^, bone augmentation, Bio-Oss^®^, hydroxyapatite, tricalcium phosphate, PLGA

## Abstract

Bone graft materials are applied in patients to augment bone defects and enable the insertion of an implant in its ideal position. However, the currently available augmentation materials do not meet the requirements of being completely resorbed and replaced by new bone within 3 to 6 months. A novel electrospun cotton-wool like material (Bonewool^®^, Zurich Biomaterials LLC, Zurich, Switzerland) consisting of biodegradable poly(lactic-co-glycolic) acid (PLGA) fibers with incorporated amorphous ß-tricalcium phosphate (ß-TCP) nanoparticles has been compared to a frequently used bovine derived hydroxyapatite (Bio-Oss^®^, Geistlich Pharma, Wolhusen, Switzerland) in vitro. The material composition was determined and the degradation behavior (calcium release and pH in different solutions) as well as bioactivity has been measured. Degradation behavior of PLGA/ß-TCP was generally more progressive than for Bio-Oss^®^, indicating that this material is potentially completely resorbable.

**Figure:**
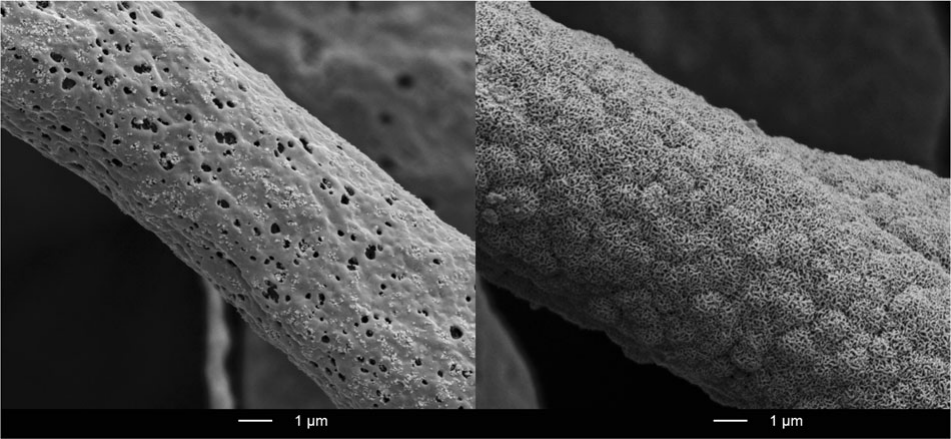
Graphical abstract

Graphical abstract

## Introduction

Dental implants are a valuable treatment option for the rehabilitation of missing teeth [1]. However, due to bone deficiencies following bone resorption in edentulous regions, placing an implant in a biomechanically appropriate restoratively-driven position is impeded. To repair the defect and to recreate bone volume, bone augmentation is performed [2–4]. Bone graft materials can provide osteogenic, osteoinductive and/or osteoconductive properties [5, 6]. Whereas “osteogenicity” generally refers to bone affinity, “osteoinduction” means that bone formation is induced even at ectopic sites and “osteoconduction” stands for bone growth onto an implant surface or into porous 3D structures [7, 8]. Biomaterials used for bone regeneration are either autografts, allografts, xenografts or synthetic materials [6].

The ideal bone graft material should be able to be completely resorbed and replaced by new bone. The ability to create new bone must be balanced with the resorption kinetics of the bone graft material to avoid losing augmentation volume or generate inflammation initiated by a foreign body response [9, 10].

Not only the chemical composition but also the 3D-morphology and in particular the presence of open pores in bone graft substitutes has been shown as a requisite of great importance for repairing osseous deficiency, favoring the osteoconduction through vascularization and osseous growth also inside the pores [11]. A resorbable bone graft material should have an adequate open porosity to allow infiltration of blood and cells. Micropores (<10 µm) assist chemical degradation of the material, mesopores (>10 µm) and macropores (>100 µm) play an important part in stabilization of the initial blood coagulum and in vascularization and integration of the graft material in the surrounded area [12–14].

Another important characteristic in bone substitutes is its biodegradability, which bases on passive chemical degradation or dissolution and cellular activity [14]. The biological characteristics of bone graft materials and their regeneration mechanism are influenced by their porosity, surface geometry, chemical composition and crystallinity [15, 16].

Numerous bone substitutes are available in dentistry. Most contain calcium phosphate ceramics that display a similar structure to the mineral phase of bone such as tricalcium phosphate (TCP Ca_3_[PO_4_]_2_) or hydroxyapatite (HA Ca_5_[OH(PO_4_)_3_]) [17, 18]. HA is the principal inorganic component of the calcified tissues in human body and has a calcium/phosphorus ratio of 1.67 [19, 20]. HA is the most stable and least soluble calcium phosphate phase [21], hence HA bone grafts can be found within an augmented site even years after implantation. HA is considered to be osteoconductive but not osteoinductive [17]. A clinically tested product containing HA derived from bovine bone is Bio-Oss^®^ (Geistlich Pharma, Switzerland) [22, 23]. However, for xenogenic bone graft materials, there is still a remaining risk of infectious disease transmission (bovine spongiform encephalopathy and Creutzfeldt–Jakob disease) and ethical or religious concerns may be raised [24].

TCP is a bone graft substitute with a high biocompatibility, favorable resorption properties and osteoconductivity [25, 26]. TCP has a Ca/P ratio of 1.5 [19, 21]. It can appear in two crystalline phases: A high-temperature rhombohedral modification (α-TCP) and a low-temperature monoclinic modification (β-TCP) [26, 27]. Partial amorphous β-TCP has been demonstrated to have an increased in vivo osteoconductivity compared to HA and an increased biodegradability compared to crystalline β-TCP [18].

Furthermore, porous biphasic calcium phosphates consisting of 40% resorbable ß-TCP and 60% non-degradable HA show successful clinical performance [28] due to the fast degradation and bone stimulation of the ß-TCP phase and the preservation of bone volume by HA [29].

A synthetic bone substitute has been developed at the Swiss Federal Institute of Technology, Zurich, Switzerland. The so-called “Bonewool^®^” consists of mechanically formable, biodegradable poly(lactic-co-glycolic) acid (PLGA) fibers with incorporated amorphous β-TCP nanoparticles, thus providing a cotton wool-like consistency [30–32]. The composite material therefore is easy to shape and thus facilitates the surgical procedure [32]. PLGA is a well-established biodegradable synthetic polymer for scaffolds in tissue engineering [33–36]. PLGA is a family of Food and Drug Administration-approved polymers, which are physically strong, biocompatible and have shown a large potential as a drug delivery carrier [33, 35].

Bonewool^®^ may therefore combine properties such as flexibility and biodegradability of the PLGA polymer and the stability and high bioactivity of β-TCP [31]. Although Bonewool^®^ has been investigated in pre-clinical trials, no data on the in vitro degradation behavior, calcium release and prolonged bioactivity testing in comparison with a clinically established control has yet been published. The aim of the present study was to evaluate the degradation process of this novel PLGA/TCP material compared to a standard HA material.

## Materials and methods

A bone graft material with a cotton-wool like appearance (BW, Bonewool^®^, Zurich Biomaterials LLC, Zurich, Switzerland) was compared to bovine hydroxyapatite granules that are considered a gold standard material in dentistry (BO, Bio-Oss^®^, Geistlich Pharma AG, Wolhusen, Switzerland) [22, 23, 30, 37] (Table 1). According to [30, 31], BW consists of partially amorphous β-tricalcium phosphate (β-TCP) particles incorporated into electrospun poly(lactic-co-glycolic acid) (PLGA) fibers with a PLGA/β-TCP ratio of 60/40 wt%. The β-TCP particles are prepared by flame spray synthesis and with a grain size in the submicron range [30–32]. The electrospinning solution was prepared using chloroform (Riedel-de-Haën, Ph. Eur.) as solvent and Tween20 (Polysorbate20, Fluka, Ph. Eur.) as surfactant [30]. BO is a xenograft derived from deproteinized and sterilized bovine bone with particle size of 250 µm to 1000 μm and an open porosity of around 80%, determined by µCT [38]. The organic components of the bone are removed by chemical and thermal treatment (<350 °C), thus resulting in an inorganic HA phase [39].

**Table 1:** Bone graft materials tested in the present study

Code	Material	Origin	Product name	Manufacturer
BW	40 wt% amorphous tricalcium phosphate particles incorporated into 60 wt% electrospun poly(lactic-co-glycolic acid) fibers	Synthetic	Bonewool^®^	Zurich Biomaterials LLC, Zurich, Switzerland
BO	Hydroxyapatite particles	Bovine	Bio-Oss^®^	Geistlich Pharma AG, Wolhusen, Switzerland

### Material composition

#### Amount of organic fraction

The amount of organic fraction was assessed by Loss On Ignition (LOI) method. Ten samples of 4.5 g each bone graft material were oven-dried (EWL Type 5626, KaVo Dental GmbH, Biberach, Germany) to constant weight (24 h at a temperature of 105 °C). Samples were first weighed (m_1_) and subsequently heat-treated in a muffle furnace at a temperature of 500 °C for 1 h (heating rate: 40 °C/min, holding times: 10 min at 90 °C, 2 min at 200 °C, 1 h at 500 °C, cooling rate: 31.5 °C/min). Afterwards, crucibles were moved to a desiccator to cool to room temperature and again weighed 10 min after removal from the furnace (m_2_). LOI was calculated using the following equation:

$${{{\mathrm{LOI}}}} = \left( {{{{\mathrm{m}}}}_1-{{{\mathrm{m}}}}_2} \right)/{{{\mathrm{m}}}}_1.$$

Unused bone graft materials and samples after LOI were visualized by scanning electron microscopy (SEM) (ESEM XL-30, Philips, Eindhoven, the Netherlands) using SE and BSE detector. Specimens were sputtered with a 20 nm layer of gold for conductivity and analyzed at 10 kV.

Additionally, a thermogravimetric analysis (TGA) was performed with one sample of 4.5 g per material (STA 449 C Jupiter, Netzsch, Selb, Germany) from 25 °C to 1000 °C, applying a heating rate of 5 K/min.

#### X-ray diffraction analysis (XRD)

X-ray diffraction analysis (D2 Phaser, Bruker, Karlsruhe, Germany) was performed to identify crystalline phases by analyzing the diffraction patterns. Samples were pestled and inserted into a holder. Crystalline phases were identified by comparison with the International Centre for Diffraction Data (ICDD) database (PDF-2, 2010).

### Degradation behavior

#### Calcium release

A total of 0.15 g of each bone graft material was added to 10 mL of distilled water in a test tube. The samples were stored at 37 °C for 1 d, 4 d, 9 d, or 16 d, respectively. When the end point was reached they were centrifuged at 1000 rpm (Ecco Centrifuge Type 25 S, Conatz & Co., Berlin, Germany) for 5 min. The supernatant solution was obtained and the concentration of dissolved calcium ions was measured (LAQUAtwin B-751 Ca^2+^ compact water quality meter, HORIBA Scientific, Kyoto, Japan) after calibration using two standard solutions of 150 ppm and 2000 ppm.

#### Evaluation of pH value

0.05 g of each material (*n* = 3) were added to either 3 ml of distilled water, phosphate buffered saline (Dulbecco’s phosphate buffered saline, Merck, Darmstadt, Germany) or human blood serum, respectively and stored at 37 °C. After different time intervals (1 h, 3 h, 6 h, 24 h, 48 h, 7 d, 12 d, 14 d, 21 d and 28 d) pH value of each sample was measured with a pH meter (Orion 330 PerpHecT LogRmeter, Thermo Scientific, Waltham, USA) at a constant temperature of 22 °C. The pH meter was calibrated with standard solutions of pH 6.87 and pH 9.18 (WTW GmbH, Weilheim, Germany) prior to the measurements. The tip of the cleaned electrode was always placed in the same position, 1 mm above the material without stirring. After each measurement, the electrode was carefully rinsed with distilled water and dried with absorbent paper to eliminate material residues that could interfere with the next readings. In parallel, the pH value of the respective blank solution was obtained (*n* = 3). The change in pH value (∆pH) of the sample solutions was calculated as difference between the pH value of the sample solution and the pH value of the blank solution at the respective intervention time.

#### Bioactivity

To test bioactivity of BW and BO, the weight change after storage in a physiological environment similar to simulated body fluid (SBF) for different time intervals (12 h, 24 h, 7 d and 14 d) was recorded. A weight change is initiated by ions dissolving into the aqueous solutions (mass loss) or precipitation of HA or other compounds on the surface of the samples (mass gain). For the SBF solution, cell culture medium was prepared from Dulbecco’s Modified Eagle Medium (DMEM) powder (Sigma–Aldrich GmbH, Buchs, Switzerland, catalog number: d2902) with sodium bicarbonate added and sterile filtered. The pH of the SBF was adjusted to the physiological pH of 7.4 with 1 M HCl. Prior to immersion, the materials were dried at room temperature in a desiccator for 24 h and plasma cleaned for 2 min with oxygen plasma at a RF-level of 29.6 W (PDC-32G Harrick, oxygen purity 99.9995%, Carbagas, Gümligen, Switzerland), to ensure sterile conditions and high wettability. Approximately 0.3 cm^3^ of each bone graft material was weighed and subsequently immersed in 20 ml SBF for different time periods (12 h, 24 h, 7 d and 14 d) at 37 °C. Every 24 h the tubes were shaken slightly to ensure a homogeneous distribution of the ions around the specimens. Additionally, 10 ml medium was changed every 48 h to ensure constant ion concentrations. Before the medium was changed, the samples were centrifuged (2 min at 2500 rpm) to ensure that no granules were removed during exchange of the medium. After each time interval the samples were centrifuged, SBF was removed, samples were rinsed with nanopure water, and dried in a desiccator for 48 h to a constant weight. The dried samples were weighed again and the weight change was calculated. Statistical differences were calculated using students’ t-test (α = 0.05). Afterwards, samples were combusted according to the LOI method and XRD-analysis was performed to identify the precipitations’ crystalline phases. Samples before and after storage in SBF for 14 d were analyzed with SEM (FEI Nova Nano SEM230, Thermo Fisher Scientific, Darmstadt, Germany) using SE and BSE detector at 5 kV.

## Results

### Material composition

#### Amount of organic fraction

**Fig. 1:**
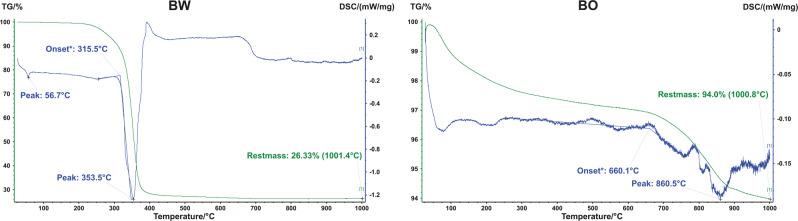
Thermogravimetric Analysis (TGA, green) and Differential Scanning Calorimetry (DSC, blue): TGA mass loss onset and DSC peaks for bone graft materials BW (left) and BO (right)

LOI (500 °C) indicating the amount of organic fraction content for BW was 75.6 ± 1.9 wt% which was significantly higher than 2.3 ± 0.4 wt% for BO (*p* < 0.001). TGA (1000 °C) revealed that for BW most of the mass was degraded at temperatures between 315 °C to 400 °C with a peak at 353.5 °C. The TGA-derived remaining inorganic mass of 26.3 wt% confirmed the LOI measurements (Fig. 1). For BO, mass loss in TGA experiments due to the combustion of the organic fraction occurred between 660 °C and 900 °C with a peak at 861 °C. The remaining inorganic mass of 94.0 wt% is therefore also consistent with the low organic fraction determined by LOI.

**Fig. 2:**
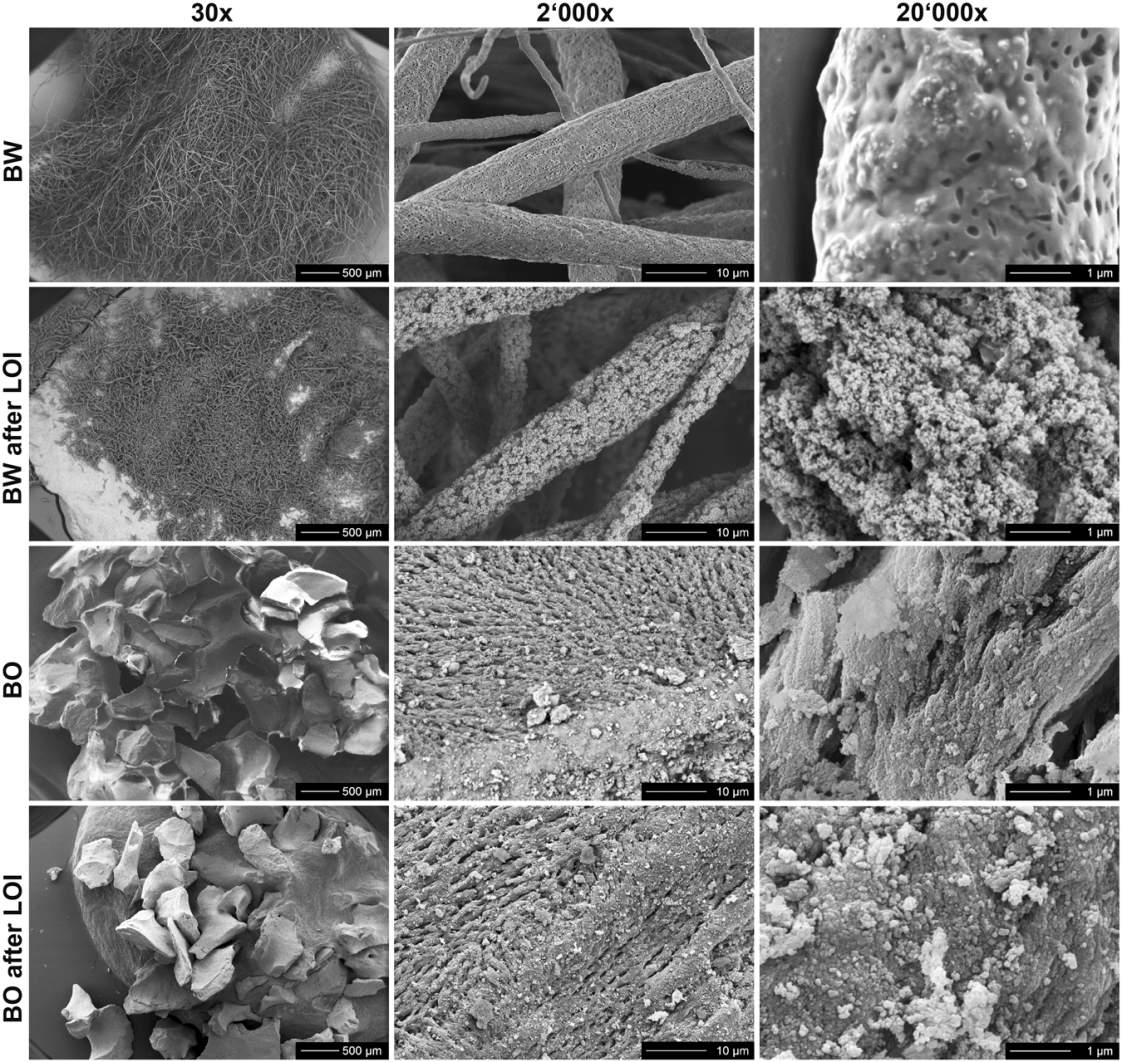
SEM images of bone graft materials BW and BO before and after Loss Of Ignition (LOI 500 °C) analysis (30x magnification bar 500 µm, 2000x bar 10 µm, 20’000x bar 1 µm)

SEM images of the bone graft materials before and after LOI analysis (500 °C) are displayed in Fig. 2. BW displayed fibers with a length of several micrometers, a diameter between 1 and 5 μm and a porous, homogeneous surface morphology. At higher magnification the porous surface of the fiber is visible, which is attributed to the evaporation of the solvent chloroform during the electrospinning process. After LOI, fibers displayed a crumbly, micro-structured surface. BO displayed granules of around 500 µm with macropores between 10 μm and 100 μm in diameter and micro-structures in the submicron range with no visible change in structure after LOI.

#### XRD

**Fig. 3:**
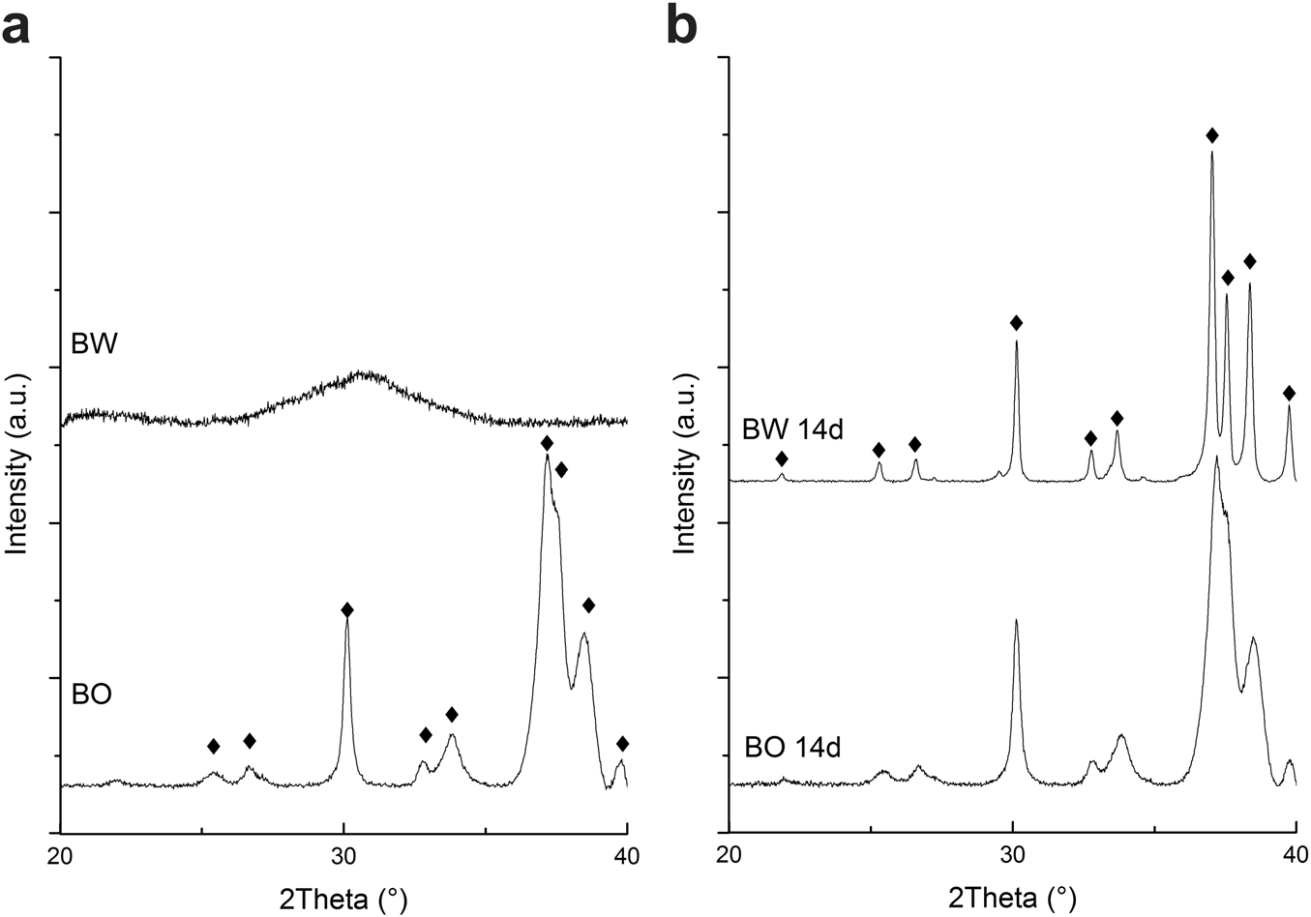
X-ray diffraction of bone graft materials BW and BO. The spectra are displayed of the materials **a** as-received and **b** after immersion in SBF during 14 d and Loss Of Ignition (LOI 500 °C) analysis. Diamond symbols indicate the HA phase

XRD-analysis of BW displayed a broad peak of partially amorphous β-TCP (Fig. 3a top) and is in agreement with previously published results [32] and PDF 00-009-0169 TCP (Whitelockit). The diffractogram of BO (Fig. 3a bottom) revealed broad reflections typical for partially amorphous HA with marked HA-peaks according to PDF 00-009-0432 HA.

### Degradation behavior

#### Calcium release

**Fig. 4:**
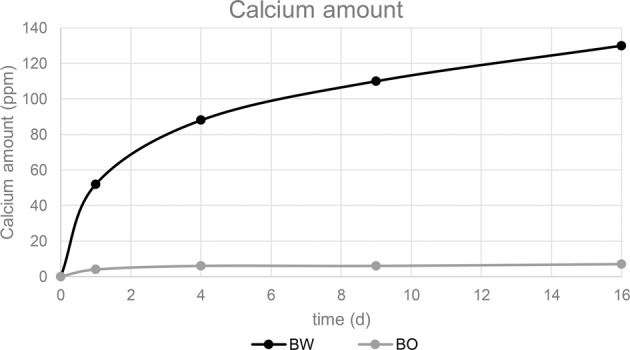
Cumulative calcium release in ppm after storage of bone graft materials BW and BO in distilled water

The cumulative calcium release of BW steadily increased from 1 d to 16 d up to 130 ppm (Fig. 4). Calcium release of BO was substantially lower, raised to 7 ppm within the first 4 d and then remained stable up to 16 d.

#### Evaluation of pH value

**Fig. 5:**
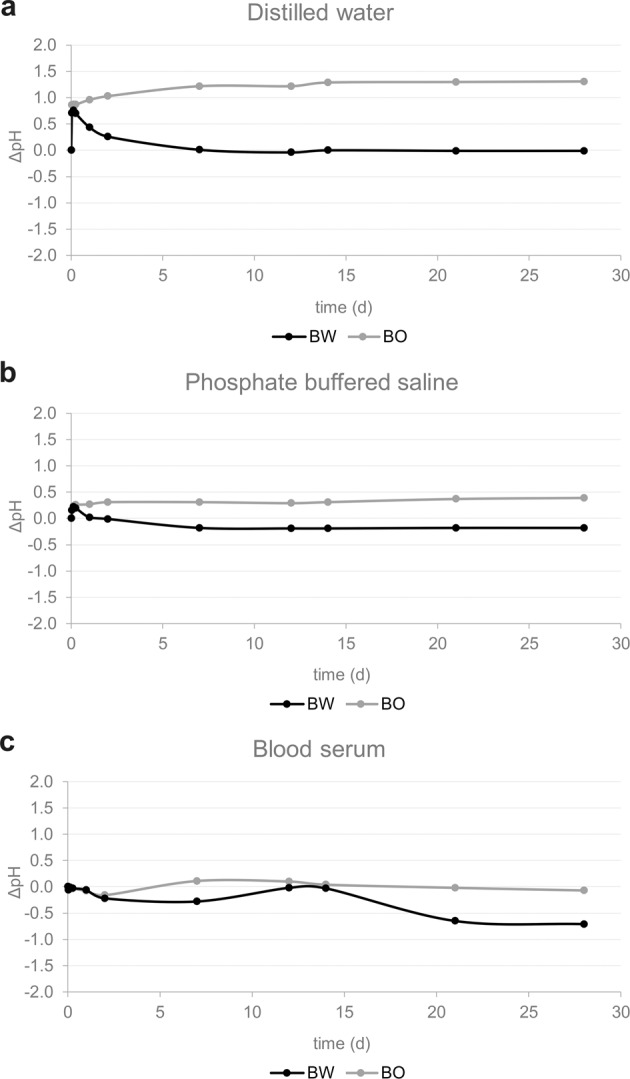
ΔpH of bone graft material BW and BO stored in **a** distilled water, **b** phosphate buffered saline and **c** blood serum for the respective time intervals

The difference in pH to the respective storage medium (ΔpH) is displayed in Fig. 5. For BW in distilled water, an initial pH increase was observed after immersion that was again neutralized within the first days (Fig. 5). A similar effect but less pronounced was observed for BW in phosphate buffer solution. For BW in human blood serum a fluctuating curve with a slight broad peak after 14 d that then turned into a lower pH was recorded. For BO in distilled water, pH slightly increased after immersion and remained constantly elevated after 7 d. Again, the same effect but less pronounced was observed when BO was placed in phosphate buffer solution. When BO was immersed in human blood serum, a fluctuating curve with a vague peak after 7 d that was buffered later is visible.

#### Bioactivity

**Fig. 6:**
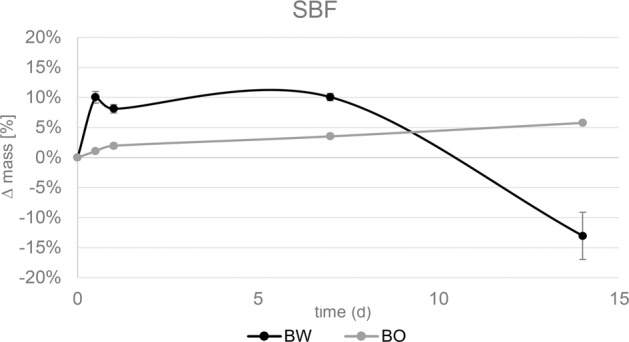
Relative mass change of bone graft materials BW and BO after storage in simulated body fluid (SBF) for the respective time intervals

**Fig. 7:**
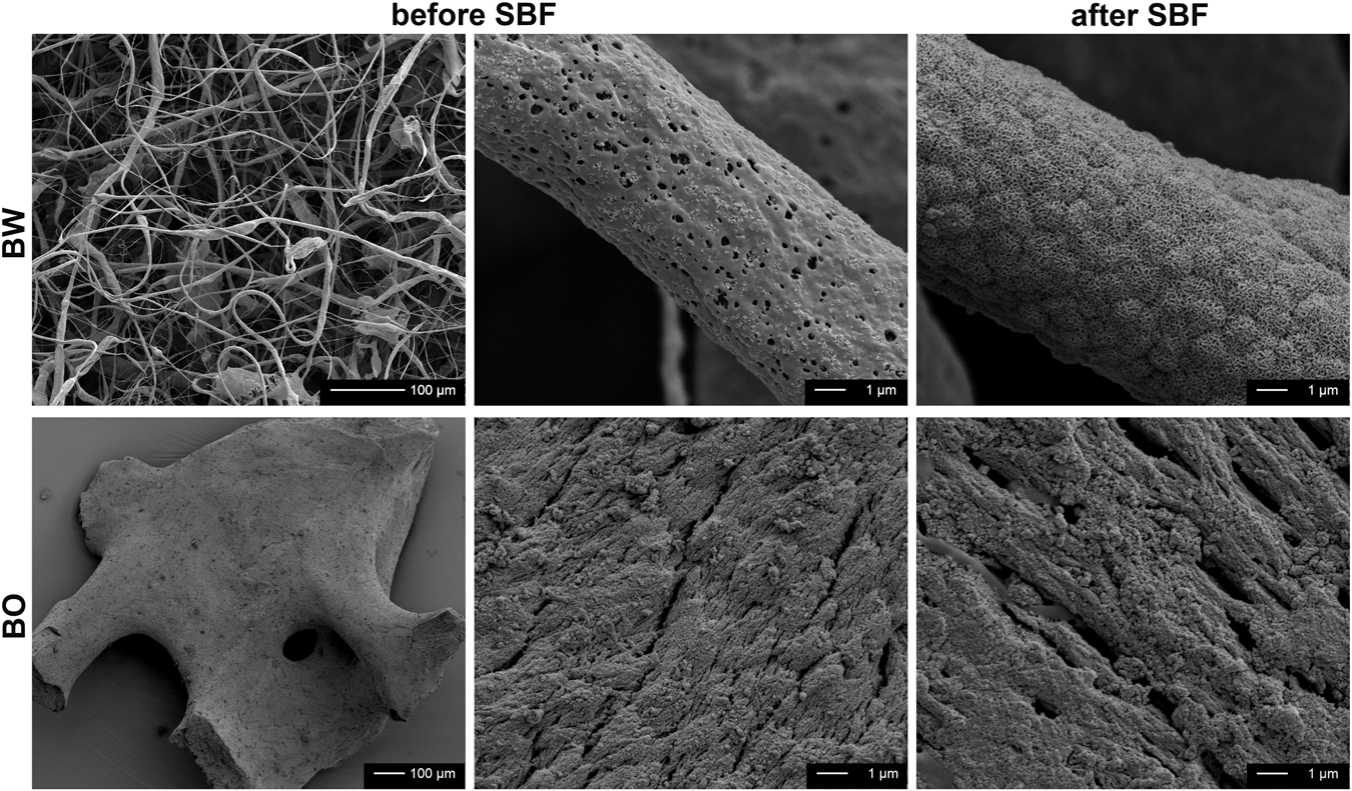
SEM images of bone graft materials BW and BO before and after storage for 14 d in SBF. β-TCP in BW and HA in BO acted as nucleation sites for HA formation as a sign of bioactivity

The weight change in % of bone graft samples BW and BO placed in SBF for different time intervals is displayed in Fig. 6. A statistically significant mass gain of 10.0% ± 1.0% compared to the initial sample mass was measured for BW after 12 h of immersion in SBF due to precipitation (*p* < 0.001). XRD-analysis of combusted samples revealed the presence of HA according to PDF 00-009-0432 HA (Fig. 3b top). SEM images show the nucleation of HA on the surface of BW and BO (Fig. 7). After 24 h, weight of BW was slightly but not significantly decreased compared to the results after 12 h to 8.1 ± 0.7% in reference to the initial sample mass (*p* = 0.286). The slight mass loss may be contributed to a degradation of PLGA or TCP. No difference in weight gain was measured after 7 d of immersion (10.1 ± 0.6%) compared to after 12 h (*p* = 0.981). A significant weight loss of −13.0 ± 3.9% of the initial sample mass was detected after 14 d (*p* < 0.001).

BO revealed a statistically significant (*p* < 0.001) mass gain between start and every single time point. After 14 d, the mass gain was 5.8 ± 0.3% of the initial sample mass.

XRD-analysis of the BO specimens after the respective immersion time did not show any differences (see Fig. 3b bottom). No other peaks were visible after immersion, indicating that only HA is found on the surface. SEM images of BW and BO display the formation of precipitates on the surfaces as a sign of bioactivity (Fig. 7).

## Discussion

The present study compared the chemical composition and degradation process of a cotton wool-like PLGA/TCP composite material (BW) to a bovine derived HA material for bone augmentation (BO). The inorganic components of both materials, β-TCP and HA, are clinically established bone substitute materials with profound evidence available [16]. For PLGA as a relatively new material clinical evidence in dental medicine is still very limited [36] and only cell and in vivo studies for the electrospun BW material [30–32] are available.

The fibers of BW presented a length of several microns, a diameter of 1–5 μm and an open-porous surface. This material meets the structural requirement of scaffolds for bone replacement that should display interconnected porosities of 50 to 90% to enhance tissue growth and vascularization [30, 40, 41]. The BO material itself consists of granules 500 µm in size with a rough surface containing macro- and micropores. In general, the presence of open pores increases the specific surface area and the number of nucleation sites of the bone graft material which promotes osteoconduction [11]. The application of large BW augmentation volumes with several millimeters in size could be advantageous, because it has been shown that large size particles (1–2 mm) are able to generate 1.4 times higher volume in sinus augmentation than smaller granules (0.25 to 1 mm) [42].

LOI and TGA revealed that BW is composed of 73 wt% organic phase that is degraded at temperatures between 315–400 °C while BO is >95 wt% inorganic and degrades at a temperature of 660–900 °C. Hence, BW may be composed rather of 70 wt% than of 60 wt% PLGA as implied by the producer [30, 31]. The weight loss of BW observed in ﻿LOI method is consistent with the result of scanning electron microscopy after LOI, where a distinct structural decomposition was observed. Since BO consists of deproteinized bovine bone, only residues of organic components might be present explaining the small amount of organic phase residue.

XRD revealed that BW is based on partial amorphous β-TCP while BO is based on HA, confirming previous findings [30, 43]. The solubility of calcium phosphates depends on the ratio﻿﻿ Ca/P and, for the same composition, it is affected by the degree of crystallinity: Less crystalline materials are more soluble [15, 44]. Therefore, it is expected that partial amorphous β-TCP with a Ca/P ratio of 1.5 [19, 21] degrades faster in BW than HA with a Ca/P ratio of 1.67 [45] in BO.

Degradation behavior of the materials was determined by analyzing calcium ion release in distilled water and measuring pH value in distilled water, phosphate buffered saline and human blood serum. Calcium ions are important to support cell viability, proliferation, and differentiation of osteoblasts [46–48] via intracellular calcium signaling [49] and therefore stimulate bone regeneration. BW showed a high calcium release from day 1 to day 16 of 130 ppm in distilled water compared to BO with only 7 ppm in the same period. This high release of BW may be due to the large amount of degradable organic composition and faster hydrolysis of β-TCP in water compared to HA.

Dissolution of BW in distilled water and phosphate buffered saline resulted in an increased pH on the first day only while afterwards the starting pH was reached again. In human blood serum pH curves of BW oscillated close to the initial pH-value. The initial increase of pH in distilled water and PBS can be explained by the release of calcium hydroxide Ca(OH)_2_ and formation of OH-ions, which results in an alkaline effect. As it is known for β-TCP, immersion of BW in aqueous solutions with a pH value of >4.2 has the combined effects of dissolution and a subsequent deposition of HA [50]. This dissolution of β-TCP results in a decrease in pH value due to the creation of HPO_4_^2−^ [50].

$$4{{{\mathrm{Ca}}}}_3\left( {{{{\mathrm{PO}}}}_4} \right)_2 + 2{{{\mathrm{H}}}}_2{{{\mathrm{O}}}} \to 2{{{\mathrm{Ca}}}}_5{{{\mathrm{OH}}}}\left( {{{{\mathrm{PO}}}}_4} \right)_3 + 2{{{\mathrm{Ca}}}}^{2 + } + 2{{{\mathrm{HPO}}}}_4^{2 - }$$

BO displayed more steady pH curves with a slight increase of up to 0.2 (blood serum), 0.4 (PBS) and 1.5 (distilled water) pH units after immersion in all three solutions. A possible reason for the more continuous and alkaline pH curves of HA (Ca_5_(PO_4_)_3_(OH)) could be that the OH^-^-concentration of HA is higher than the hydroxide of the autoprotolysis of water and therefore the pH value at the beginning is higher in BO than for BW. In human blood serum both pH curves of BW and BO slightly oscillated. However, human blood serum is a complex mixture with different substances like ions, amino acids and globulins, that may influence the pH, hence this mechanism cannot be explained within the limitations of the study. Hydrolysis of PLGA produces lactic and glycolic acid monomers that may have slightly lowered the pH of the solutions as well [51]. During wound or bone fracture healing, pH is initially lowered, leading to a local acidosis [52, 53] and triggering immune cells to the fracture site [54].

To physico-chemically estimate the bioactivity of a biomaterial, immersion in SBF is a common method that is widely used [55]. BW can be considered highly bioactive due to the precipitation of 10 wt% HA occurring already after 12 h. However, BW at the same time seems to simultaneously degrade rather fast in SBF, meaning after 24 h, the specimen mass gain marginally decreased from 10 wt% to 8 wt% indicating that the decomposition of the (polymer and ceramic) PLGA/β-TCP material was faster than precipitation of HA. A previous study found a mass gain of BW of 18 wt% after 2 days and also HA precipitation [30]. After 7 d the net increase of mass is still around 10 wt%, and represents a superimposition of PLGA/β-TCP degradation and simultaneous HA deposition. After 14 d, decomposition of PLGA and β-TCP must have been severely higher than the HA-precipitation resulting in a net mass loss of −13 wt% compared to the initial mass. This finding is supported by a previous study where the inorganic component of PLGA/β-TCP after storage in SBF increased constantly [31]. Our measurements of the released Ca amount confirm the trend towards persistent degradation.

On BO, precipitation of HA constantly increased over time and reached 6 wt% after 14 d. The fast degradation of BW due to its chemical composition, its high porosity and consequently high surface may be concerning because if passive chemical dissolution is too fast, bone substitutes disappear before new bone formation occurs. For β-TCP based materials, chemical dissolution, possibly favored by a high cell metabolism in the particles, has been identified as the predominant cause of β-TCP degradation [56]. Hence, it is of high importance that chemical dissolution of a bone substitute and bone growth have to be balanced to maintain the achieved augmentation volume. For complete bone substitute resorption, 3–6 months is an appropriate interval considering the speed of bone remodeling and new bone formation [57–59].

Although the in vitro behavior of BW and BO varies regarding composition and degradation behavior, a preclinical study in a rabbit model [30] that compared both materials revealed a comparable outcome. However, new formed bone within BW displayed a finer, more spongeous appearance compared to the more solid cortical bone formation with BO [30]. Further studies are necessary to understand the correlation between the dynamic of degradation and bone apposition in vitro and in vivo.

## Conclusion

The most significant hydroxyapatite precipitation was observed on the cotton-wool like PLGA/β-TCP material compared with the bovine derived hydroxyapatite material. The increased calcium release of Bonewool^®^ compared with Bio-Oss^®^ might stimulate bone regeneration by affecting intracellular calcium signaling of osteoblasts. Dissolution of PLGA/β-TCP in either distilled water, PBS or human blood serum tended to lower pH values compared with the hydroxyapatite material, which may attract immune cells to the implant site. Hence, Bonewool^®^ displays promising properties to accelerate HA deposition, osseoinduction and osseointegration.

## Data Availability

Data available on request from the authors.
